# The policy-practice gap: describing discordances between regulation on paper and real-life practices among specialized drug shops in Kenya

**DOI:** 10.1186/1472-6963-14-394

**Published:** 2014-09-16

**Authors:** Francis Wafula, Timothy Abuya, Abdinasir Amin, Catherine Goodman

**Affiliations:** KEMRI/Wellcome Trust Research Programme, P. O. Box 43640–00100, Nairobi, Kenya; Population Council, Nairobi, Kenya; ICF International, Nairobi, Kenya; London School of Hygiene and Tropical Medicine, London, UK

**Keywords:** Regulation, Pharmaceutical Services, Drug Shops, Kenya

## Abstract

**Background:**

Specialized drug shops (SDSs) are popular in Sub-Saharan Africa because they provide convenient access to medicines. There is increasing interest in how policymakers can work with them, but little knowledge on how their operation relates to regulatory frameworks. This study sought to describe characteristics and predictors of regulatory practices among SDSs in Kenya.

**Methods:**

The regulatory framework governing the Kenya pharmaceutical sector was mapped, and a list of regulations selected for inclusion in a survey questionnaire. An SDS census was conducted, and survey data collected from 213 SDSs from two districts in Western Kenya.

**Results:**

The majority of SDSs did not comply with regulations, with only 12% having a refrigerator and 22% having a separate dispensing area for instance. Additionally, less than half had at least one staff with pharmacy qualification (46%), with less than a third of all interviewed operators knowing the name of the law governing pharmacy.

Regulatory infringement was more common among SDSs in rural locations; those that did not have staff with pharmacy qualifications; and those whose operator did not know the name of the pharmacy law. Compliance was not significantly associated with the frequency of inspections, with over 80% of both rural and urban SDSs reporting an inspection in the past year.

**Conclusion:**

While compliance was low overall, it was particularly poor among SDSs operating in rural locations, and those that did not have staff with pharmacy qualification. This suggested the need for policy to introduce levels of practice in recognition of the variations in resource availability. Under such a system, rural SDSs operating in low-resource setting, and selling a limited range of medicines, may be exempted from certain regulatory requirements, as long as their scope of practice is limited to certain essential services only. Future research should also explore why regulatory compliance is poor despite regular inspections.

## Background

Specialized drug shops (SDSs) are key players in providing access to medicines and other health services in developing countries. Their influence is reflected in the growing interest to include them in the provision of essential services, including treatment of malaria [1, 2], diarrhea [3], respiratory tract infections [4], and non-infectious diseases such as hypertension and dyslipidemia [5, 6]. The term ‘specialized drug shops’ refers to shops that sell medicines and related commodities [7]. The scope of SDSs varies across countries, but will usually include registered and unregistered pharmacies and drug shops [8]. Because SDSs are a component of the health care system, they are governed by regulations in ways that are similar to other health service providers.

Regulation of private health providers prescribes minimum entry conditions, quality standards, and operational requirements [9, 10]. For SDSs, regulations typically outline personnel qualification requirements, structural design features for the premises, minimum equipment and material requirements, and provisions for good medicine storage and dispensing practices [11–13]. However, studies have shown SDSs to be poor regulatory compliers in developing countries. Among the unlawful practices reported are the sale of unauthorized medicines, dispensing without prescription, and failing to comply with requirements for personnel and premises [14–16]. Such regulatory infringements can affect quality of care and possibly even endanger the lives of clients, thus undermining the public health importance of SDSs.

In Kenya the main legislation governing the retail pharmaceutical sector is the Pharmacy and Poisons Act of 1959, though several other regulations are also relevant (Table 1) Table 2 summarizes key regulatory provision in the retail sector.

**Table 1:** **Rules and regulations governing the retail pharmaceutical sector in Kenya**

Legislation/regulation/rule	Main purpose	Front-line enforcers
The Pharmacy and Poisons Act (Cap 244)	Govern pharmacy profession, and manufacture, supply and use of medicines	Pharmaceutical inspectors
The Public Health Act	Govern all aspects of public health, including medicines	Public health officers
The Food, Drugs and Chemical Substances Act	Ensure safe provision of foods, drugs and chemical substances	Public health officers
The Local Government Act	Govern local authorities, including licensing local service providers	Local council inspectors
The Narcotic Drugs and Psychotropic Substances Act	Control possession and trafficking of narcotic drugs and psychotropic substances	All types of inspectors (includes public health officers, pharmaceutical inspectors)
Anti Counterfeit Act	Prevent counterfeiting of medicines and related commodities	All types of inspectors
Guidelines for Registration of Premises	Outline minimum requirements for issuance of wholesale and retail licenses	Pharmaceutical inspectors
Guidelines for Good Wholesaling and Retailing Practice	Outline minimum requirements for the daily operation of a retail or wholesale pharmacy	Pharmaceutical inspectors
Continuous Professional Development points system	Assess practitioners for a minimum score with regard to professional knowledge	Pharmaceutical Society of Kenya Training Department

**Table 2:** **Rules governing the retail pharmaceutical sector in Kenya**

Area regulated	Specific rules
Structure-related regulations	▪ → Premises must be registered
	▪ → Annual premise license must be in place
	▪ → Licenses must be displayed on the wall
	▪ → Premises must comply with structural requirements for pharmacy practice:
	- → Premises must have construction of permanent nature
	- → Premises must be well lit and ventilated
	- → Premises must have a separate dispensing area of minimum size 8 by 10 feet
	- → Premises must have water, toilet facilities and lighting; proper storage area for medicines
Personnel and practice related regulations	▪ → Personnel must have proper qualification and registration
	▪ → Annual practice license must be in place
	▪ → Dispensing prescription medicines only with a valid prescription
	▪ → Medicines sold must be properly labeled
	▪ → Advertising of medicines and services not allowed without due permission
	▪ → SDSs must not stock other household commodities alongside medicines
	▪ → Records must be kept for the sale of prescription medicines
Equipment and materials related regulations	▪ → The premises should have basic equipment, including a refrigerator and a narcotics cupboard
	▪ → The premises should have reference materials such as Martindale, reference showing drugs available in the Kenyan market, and the Pharmacy and Poisons Act

Regulation is overseen by the Pharmacy and Poisons Board (PPB), Kenya’s medicines regulatory authority. Regulations are enforced by pharmaceutical inspectors and public health officers (PHOs), whereas enforcement of professional ethics is overseen by the professional bodies representing pharmacists (degree holders) and pharmaceutical technologists (diploma holders). Regulations are accompanied by penalties for non-compliers, ranging from fines and suspension to prison sentences. In Kenya, only SDSs operated by registered pharmacists or pharmaceutical technologists are recognized by law. Both cadres are allowed to dispense prescription only medicines and over the counter medicines. However, it is widely known that a large proportion of SDSs are not operated by the two cadres; for this reason, we use the term SDSs to refer to pharmacies that are operated by qualified and unqualified personnel.

SDSs rank among the largest category of health service providers in Kenya. In 2008 there were roughly 5,300 public health facilities [17], while the number of licensed and unlicensed SDSs was estimated at about 6,000 [18]. Previous studies have shown that 26 - 69% of the Kenyan population visit retailers as the first point of contact for common illnesses such as fever [19–22].

Although studies have raised concern over the quality of services provided by pharmacies in Kenya [18, 23, 24], there is a lack of evidence on the relationship between regulation on paper and practices among licensed and unlicensed SDSs. Understanding this relationship is important both in terms of the public health implications of regulatory non-compliance, and in understanding the regulatory environment and likely implications for relationships between inspectors and SDS. This study sought to document levels of compliance with selected regulations, and to identify risk factors for non-compliance.

## Methods

### Study sites and population

**Figure 1:**
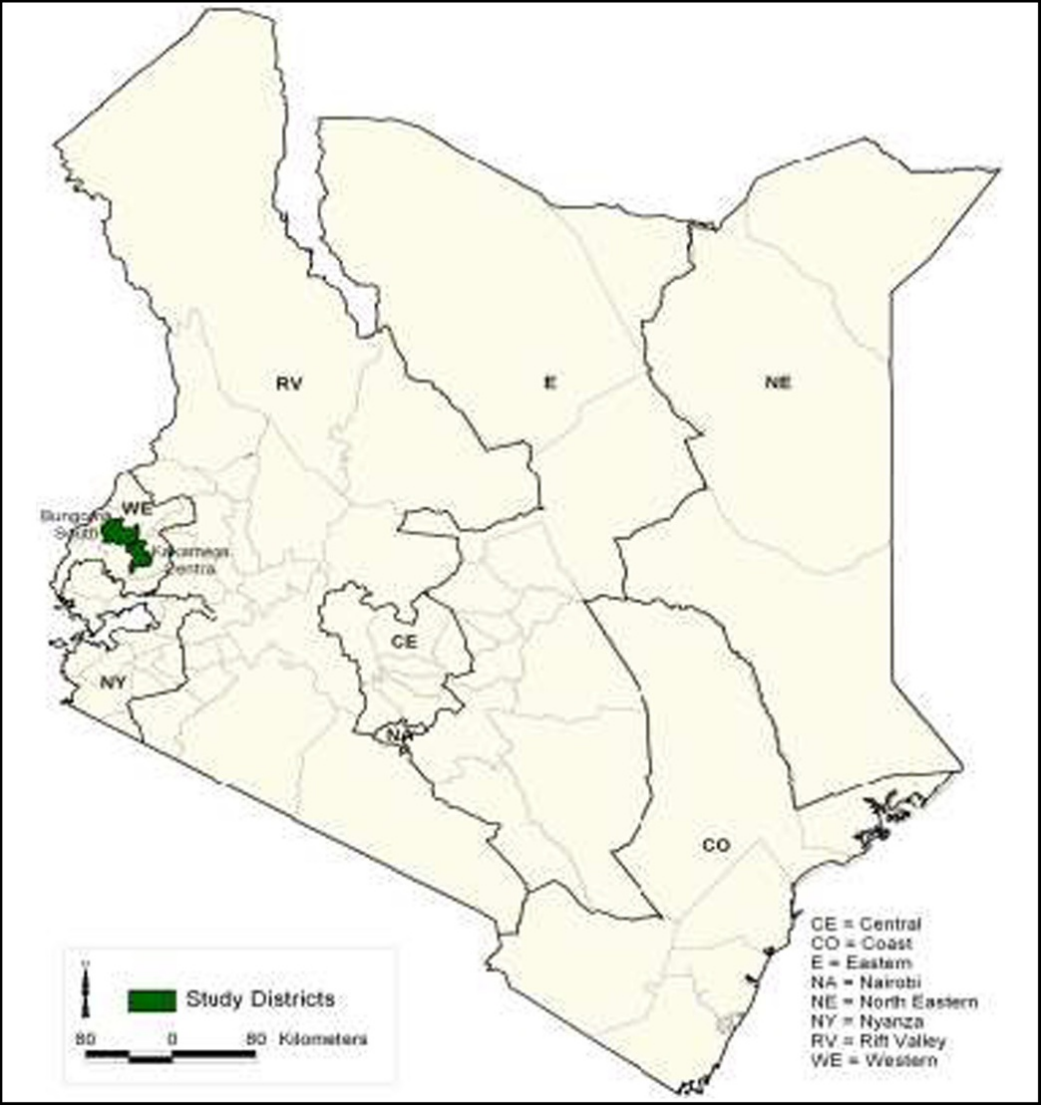
**Map of Kenya showing study districts.**

Data were collected in 2 districts from the western region of Kenya; Bungoma South and Kakamega Central (Figure 1). The two are among the most densely populated districts in Kenya, with a population density of 602 (Bungoma) and 723 (Kakamega) people per km^2^, against a national average of 68 [25, 26]. These districts were selected as SDSs were known to be numerous in the Western Region, and therefore of high policy importance. Both districts also have urban and rural settings, allowing comparison of outcomes by location.

The commonest causes of morbidity and mortality in the region are malaria, diarrhoea, respiratory diseases, and HIV/AIDS [25, 26]. The two districts have similar socioeconomic status, with national statistics indicating that 51% and 54% of individuals live below the poverty line in Bungoma and Kakamega, respectively (based on estimates of expenditures on minimum food and non-food item requirements) [27]. Both districts have at least one public hospital and several private nursing homes and public primary care facilities. As a result, over 90% of the population lives within 5 km of a health facility (personal communication from KEMRI GIS Department colleagues). However, the facilities suffer from low staffing levels, with doctor-patient ratios of 1:26,613 (Bungoma) and 1:20,835 (Kakamega), way below the WHO recommended figure of 1:1,000 [25, 26].

### Sampling and data collection procedures

Universal sampling was used, where all identified SDSs were included in the study. Shops were identified through a census, and data collected using an SDS survey. The census was necessary because information held by the health authorities on the numbers and location of SDSs was likely to be incomplete and outdated. During the census, shops were identified in 3 main ways; first, the field team was recruited from the study areas, and was able to identify some SDSs; second, local public health officers (PHOs) were recruited as guides; and finally, staff working in enumerated shops was asked to identify other shops within the area.

Surveys were done between September and December 2009 by five trained field staff. Following verbal consent, the SDS survey questionnaire, previously piloted outside of the study areas, was administered to persons most involved with day to day management of the shops. As SDSs generally have staff with some formal education, the survey was administered in English. The survey tool covered staff characteristics; location and structural features of the premises; practice aspects such as the keeping of prescription records; and the range of commodities stocked.

Specific regulatory behaviors were selected for investigation using the following procedure. First, the diverse legal and regulatory framework governing the pharmaceutical sector was mapped. This entailed identifying relevant legislation and regulations from the medicines regulatory authority (PPB), and guidelines from the professional body representing pharmacy practitioners, as summarized in Table 1. These regulations were then classified as structure-related, equipment and material requirements, and personnel and practice related regulations. Two or three indicators were selected to represent each group, focusing on those that could be assessed objectively by our field workers, without the need to make subjective judgments or to observe consultations. Indicators selected were: the building material for the premises, and presence of a separate dispensing area to represent structure; availability of a refrigerator, a narcotics cupboard, and a prescription book to represent equipment and materials; staff qualification and knowledge of the name of the Pharmacy Act to represent personnel factors; and displaying of licenses and stocking of other household commodities to represent practice-related factors. We decided not to assess validity of licenses, as we were concerned that this could give the impression that the interview was an inspection which could have damaged rapport with the respondent and possibly exposed our field staff to aggression, given the sensitivity of this area. It was important that we did not threaten the rapport with providers, as this study formed part of a larger programme of work, involving a qualitative study of factors affecting regulatory compliance, drawing on in-depth interviews with the same retailers [28]. Moreover, inspecting a license does not in itself allow assessment of SDS status as the named individual may not be involved in day to day SDS operation.

We recognize that the regulations selected for the study may not be reflective of public health importance, and that other regulations with higher public health significance may have been omitted for logistical reasons. However, we also observe that the primary objective of the study was to measure regulatory compliance, and not the relative merit of different regulations.

### Data management and analysis

Quality checks were made by a field supervisor and the first author during data collection. Survey data were double entered into MS Access 2007, and analysis was conducted in Stata v11.0 (Stata Corporation, TX, USA). The unit of analysis was the SDS for all variables. SDSs operating within the main town were classified as urban, and those located more than 3 km from the town classified as rural.

To compare SDS characteristics across districts and urban/rural location, chi-square tests were performed. Associations between selected regulatory practices and shop and staff characteristics were explored using multivariate analysis. The district and urban/rural location variables were automatically included in the multivariate model because there were strong reasons *a priori* for expecting them to be associated with regulatory practices. As regulatory enforcement is likely to be influenced by the district health authority, it was expected that SDSs in the two districts could vary depending on the nature and capacity of staff at the district health office. Similarly, location was expected to influence regulation, as rural SDSs have previously been shown to offer lower quality of care in Kenya [24]. A stepwise selection algorithm was applied to identify other variables for inclusion, with only those variables with a univariate Wald test p-value of <0.2 being included [29, 30].

Institutional ethical approval was provided by both the KEMRI/Wellcome Trust Research Programme and the London School of Hygiene and Tropical Medicine, and national approval by the KEMRI National Ethical Review Committee.

## Results

### Characteristics and regulatory practices of specialized drug shops

A total of 130 and 94 shops were identified as operational in Bungoma and Kakamega respectively through the census. The numbers in both districts were substantially higher than expected based on projections from previous work in the region, and on estimates provided at the local health offices, which had indicated an estimated total of 110 SDSs across both districts.

The medicine retailer survey was completed in 213 of the 224 SDSs, with 11 (5%) refusing consent. The main reason for refusal was the absence of the staff-in-charge. Thirty SDSs were situated in urban locations in Bungoma (25%), with Kakamega having 35 urban SDSs (38%). The majority of SDSs had the owner working there (56 and 60% in Bungoma and Kakamega respectively).

Table 3 shows the regulatory characteristics stratified by district and rural–urban location. Shops in the two districts were similar with regard to qualification of staff, displaying of licenses, and availability of a working refrigerator. Regulations that reported the lowest compliance were the presence of separate dispensing rooms and availability of narcotics cupboards and refrigerators. Pharmacy qualified staff (pharmacists or pharmaceutical technologists) were relatively few overall (43 and 51% of SDSs in Bungoma and Kakamega), with the most common non-pharmacy cadre being nurse aides (54% of SDSs) and nurses (33%). Nurse aides are trained on-the-job to carry out a limited range of tasks, whereas nurses have diploma or degree training in nursing, and offer all nursing services. Nearly all shops reported having at least one staff member with some health-related training (defined broadly to include cadres such as nurse aides).

**Table 3:** **Regulatory characteristics of SDSs in the two districts (n = 213)**

	Total	Districts			Locations		
	n (%)	Bungoma	Kakamega	p-Value	Urban	Rural	p-Value
		n (%)	n (%)		n (%)	n (%)	
Number of shops surveyed	213	120	93	-	65	148	-
**Shop and staff characteristics**							
Premises made of bricks or stone	210 (99)	119 (99)	91 (98)	0.4	64 (98)	146 (99)	0.08
Shops with separate dispensing room	46 (22)	19 (16)	27 (29)	0.02	28 (43)	18 (12)	<0.001
Shops selling household merchandise with medicines	50 (23)	26 (22)	24 (26)	0.5	13 (20)	37 (25)	0.4
Shops displaying any licenses on the wall	119 (56)	66 (55)	53 (58)	0.7	41 (63)	78 (53)	0.1
Shops with a pharmacy-qualified staff member^1^	99 (46)	52 (43)	47 (51)	0.3	39 (60)	60 (41)	0.009
Staff knows name of main law governing pharmacy^2^	64 (30)	33 (27)	31 (33)	0.4	33 (51)	31 (21)	<0.001
**Availability of materials and equipment**							
Shops with a prescription recording book	89 (42)	58 (48)	31 (33)	0.03	37 (57)	52 (35)	0.003
Shops with a working refrigerator available	25 (12)	13 (11)	12 (13)	0.6	18 (28)	7 (5)	<0.001
Shops with a narcotics cupboard available	39 (18)	26 (22)	13 (14)	0.2	24 (37)	15 (10)	<0.001
**Frequency of regulatory inspection**							
Shops inspected within the last 12 months	182 (85)	104 (87)	78 (84)	0.6	58 (89)	124 (84)	0.3

^1^Includes pharmacists and pharmaceutical technologists, the two cadres recognized by the Kenyan law.

^2^Staff who could correctly name the ‘Pharmacy and Poisons Act’ as the main legislation governing pharmacy.

There were some differences between the districts, with more shops in Kakamega having a separate dispensing area compared to Bungoma (29 and 16% respectively, p = 0.02), and more shops in Bungoma having prescription recording books (48 and 33% respectively, p = 0.03).

Stratification by location showed urban shops to be better regulatory compliers overall, with more shops having separate dispensing areas, prescription recording books, working refrigerators and at least one staff member with pharmacy qualifications. More respondents in urban shops also knew that the Pharmacy and Poisons Act was the main legislation governing pharmacy practice in Kenya. As far as inspections were concerned, little difference was observed across the two locations or between districts, with the proportion of shops reporting at least one regulatory visit over the last year being high overall (over 80% of shops in both urban and rural locations and in both districts). Varying the 3 km threshold for the definition of “rural” in the analysis did not substantially change the allocation of SDS between rural and urban categories, as most ‘rural’ SDSs were located in small trading centres located 10–60 km from the main town.

### Predictors of regulatory practices of specialized drug shops

We examined predictors of three regulatory compliance outcomes, selected as tracers for regulatory practices (keeping prescription records), equipment requirements (availability of a refrigerator), and licensing requirements (displaying licenses within premises) (Table 4).

**Table 4:** **Univariate and multivariate analysis of predictors for selected regulatory practices (n = 213)**

		Keeping prescription records			Availability of a working refrigerator			Displaying licenses in premises		
Predictor variable		n (%)	Unadjusted OR (95% CI) p-value	Adjusted OR (95% CI) p-value	n (%)	Unadjusted OR (95% CI) p-value	Adjusted OR (95% CI) p-value	n (%)	Unadjusted OR (95% CI) p-value	Adjusted OR (95% CI) p-value
***District***										
	Bungoma	42 (35)	1.09 (0.61-1.95) p = 0.800	1.31 (0.69-2.50) p = 0.400	13 (10)	0.82 (0.35-1.90) p = 0.600	0.86 (0.30-2.47) p = 0.700	66 (55)	0.89 (0.51-1.55) p = 0.700	0.95 (0.52-1.74) p = 0.900
	Kakamega	30 (33)			12 (13)			53 (58)		
***Shop location***										
	Urban	31 (50)	2.58 (1.37-4.86) p = 0.002	1.94 (0.97-3.88) p = 0.050	18 (28)	7.71 (2.86-20.77) p < 0.001	4.14 (1.39-12.27) p = 0.010	41 (63)	1.55 (0.84-2.85) p = 0.200	0.99 (0.50-1.97) p = 0.900
	Rural	41 (28)			7 (5)			78 (53)		
***Licenses displayed in premises***										
	Yes	49 (42)	2.19 (1.18-4.08) p = 0.010	1.35 (0.69-2.67) p = 0.300	23 (19)	10.66 (2.30-49.26) p < 0.001	4.02 (0.81-20.10) p = 0.090	-	-	-
	No	22 (25)			2 (2)			-		
***Pharmacy-qualified staff***										
	Yes	49 (50)	3.71 (1.96-7.00) p < 0.001	2.70 (1.38-5.31) p = 0.004	23 (23)	16.94 (3.53-81.20) p < 0.001	6.21 (1.26-30.54) p = 0.020	69 (70)	2.95 (1.63-5.34) p < 0.001	1.95 (1.05-3.64) p = 0.030
	No	23 (21)			2 (2)			50 (45)		
***Knows name of pharmacy law***										
	Yes	35 (55)	3.52 (1.84-6.71) 001	1.98 (0.95-4.10) p = 0.060	21 (33)	17.70 (5.10-61.49) p < 0.001	4.91 (1.33-18.12) p = 0.010	51 (81)	4.94 (2.33-10.44) p < 0.001	3.63 (1.67-7.89) p < 0.001
	No	37 (26)			4 (3)			68 (46)		
***Inspection in the last year***										
	Yes	62 (35)	1.05 (0.47-2.41) p = 0.900	-		2.09 (0.46-9.44) p = 0.300	-	105 (59)	1.72 (0.80-3.73) p = 0.200	1.33 (0.59-3.01) p = 0.500
	No	10 (33)			23 (13)			14 (45)		

Staff qualification was strongly associated with keeping of prescription records, with shops having staff with a pharmacy qualification having nearly 3 times the odds of keeping the records compared to those without pharmacy-qualified staff (adjusted OR 2.70, p = 0.004) (Table 4).

Unadjusted analysis also found shop location and staff knowledge to be predictors for keeping of records (p = 0.002 and p < 0.001 respectively), but both associations weakened in the adjusted analysis (p = 0.050 and 0.060 respectively).

As noted above, availability of refrigerators was poor across SDSs overall. However, important variations emerged across shops. Adjusted analysis found strong evidence linking availability of a fridge to shop location, with shops in urban locations having 4 times the odds of having a fridge (OR 4.23, p = 0.009). Pharmacy qualification and knowing the name of the legislation governing pharmacy were also positively associated with the outcome, and with displaying of licenses within the premises (p = 0.03 and p < 0.001 respectively).

A common finding across the analysis of predictors was the weak association between a regulatory inspection in the past year and regulatory compliance.

## Discussion

This paper has presented data on regulatory practices of SDSs in terms of a selected set of requirements stipulated under the Kenyan legal and regulatory framework, and explored variation in regulatory compliance by location and other shop characteristics. Regulatory compliance was poor overall, but especially across rural shops, where the majority failed to meet requirements for staff qualification, structure, premises and equipment.

Certain methodological limitations should be noted. Although great care was taken to capture all shops by using several complementary approaches, the possibility that a few were omitted cannot be discounted. However, the fact that we found over twice as many as estimated by district health officers indicates that many of those less likely to be on official records were identified by our approach. Another potential source of bias is refusals; while these were few overall (5%), one might expect these shops to be less likely to comply with regulations. There was also risk of reporting bias, especially on items that could not be verified easily, for instance, staff qualification. The need to prioritize indicators that could be measured without making subjective judgments, observing consultations or raising suspicion that our staff were inspectors limited the study’s ability to capture a comprehensive picture of regulatory compliance.

Caution is required when extrapolating findings to other regions, as previous studies have shown the Western region to have high retail sector activity [31, 32]. Additionally, variations in the distribution of health facilities may influence retailer behaviour. Previous work has shown the Western region to have relatively good geographic access to health facilities [17], which might be expected to influence client demand and thereby dispensing practices [24].

Key aspects of regulatory compliance documented by this study include the finding that only 46% of SDS had staff with a pharmacy qualification. Kenya, like many other countries across the world, has a stringent licensure system for individuals wishing to operate SDSs, which includes 4 or 5 years pre-service training and a one-year post-training internship for pharmacists, and three years training for pharmaceutical technologists. Both cadres also require a one-off professional registration by PPB, and annual practice licenses for those practicing in their capacity as pharmacists or technologists. This stringency is a common characteristic in health care markets, and has been a subject of debate over the years [33–35].

In Kenya, year 2008 estimates put the number of registered pharmacists and pharmaceutical technologists at 2,775 and 1,680 against an estimated population of 37.5 million [18], giving a ratio of one pharmaceutical staff to 8,417 people. The problem is exacerbated by the fact that licensed individuals prefer urban locations, leaving the rural areas with even fewer personnel. This explains why staff without pharmacy qualifications were the main operators in rural SDSs. The predominance of nurse aides staffing SDSs is likely to reflect their low marketability in the formal health sector. Similar patterns have been observed in Tanzania, where the majority of staff in private drug stores were found to be nurse assistants [36].

As far as the structure of the premises and availability of materials are concerned, the majority of shops lacked a separate dispensing area, a refrigerator, a prescription recording book, and a cupboard for keeping narcotic and psychotropic substances. However, urban shops were generally better regulatory compliers compared to rural shops. Similar observations were made in Sri-Lanka, where the design and construction features of rural pharmacies were found to be poorer than those of urban shops [11]. A number of factors may explain why rural SDSs were less likely to comply with structural and equipment requirements, including lower capital injection in the business, and lower stringency from regulatory inspectors during rural visits, who may recognize that such rural shops may not need to meet all these requirements if for instance they do not sell narcotics, or medicines requiring refrigeration [8, 28].

Availability of refrigerators showed substantial variation across locations, with rural shops again performing poorly compared to urban shops. However, the availability of refrigerators was low overall (12%). Similarly, a study in Malawi found three-quarters of practitioners stocking and dispensing medicines without a refrigerator, which was against the country’s regulatory requirements [37]. In contrast, nearly all pharmacies in Sri-Lanka [11] and Pakistan [38] were found to have refrigerators, with the Sri-Lankan study reporting a 100% refrigerator availability among rural pharmacies [11]. The disparity across countries in the 2 continents with similar regulatory requirements may be a consequence of differences in perceptions of the importance of specific regulations (a refrigerator may be a more important regulatory marker in South-East Asia than in Africa), or may simply be a result of differences in the availability of electricity, or differences in the levels of technological advancement across the 2 regions.

Good regulatory compliance on the other hand, was reported with regard to the nature of materials used in constructing premises, as well as refraining from stocking non-medical commodities. Similar findings have been seen elsewhere in SSA, with two-thirds of patent medicine vendors (PMVs), and three-quarters of pharmacies selling medicines and related commodities only in Nigeria and Somaliland respectively [1, 39]. A number of factors may explain the preference for trading in medicines only, including higher profits, insufficient capital to diversify, the need to present a professional look, and strong competition in the market for non-medical commodities.

Less than a third of staff knew the name of the Pharmacy and Poisons Act with nearly all staff who got the name right working in urban locations. This is not unique to Kenya; even worse results were seen in Tanzania where only 3% of SDS owners, and 8% of dispensers knew the name of legislation governing pharmacy practice [40]. This raises two key questions: one, can providers comply with provisions of a regulation whose identity they do not appear to know? Two, what do regulatory visits entail? Are regulators required to explain the legal basis for inspections during regulatory visits, and do they?

The majority of shops reported having received at least one regulatory visit over the past year, a frequency that was similar to that reported in a separate assessment conducted in Nairobi [18]. Frequency of regulatory visits varied across Sub-Saharan African countries, with only a third of PMVs in Nigeria reporting visits over the last 2 years, while the majority of drug shops in Tanzania had received visits over the last year [41, 42]. Where inspection frequencies were low, lack of human and financial resources was commonly cited as the reason [43, 44]. Although the relationship between frequency of inspections and regulatory compliance has rarely been studied in low-income settings, lower inspection frequencies are often blamed for poor regulatory compliance [45]. However, this was not the case in this study.

It is worth noting that not all practices showed variations across locations. The practice of displaying licenses, for instance, did not vary significantly across rural (53%) and urban areas (63%). Low compliance to similar regulatory requirements has also been reported in Tanzania, where only a third of shops were found displaying the Pharmacy Board permit, and in Sri-Lanka, where 57% did not display licenses as required by law [11, 42].

Aside from location, staff qualification was an important predictor of regulatory compliance. Shops with qualified staff were more likely to display licenses, and have prescription records and a refrigerator. Besides bearing a strong association with displaying of licenses, knowledge of the name of the Pharmacy and Poisons Act was associated with availability of a working refrigerator and the practice of keeping prescription records. One would also expect licensed pharmacies to be more compliant but a significant association was not found keeping prescription records or having a working refrigerator. This could reflect the limitations of this indicator as a proxy for having a valid licence. It is likely that some displayed licenses were outdated, or belonged to individuals not involved in the day to day operation of the business. On the other hand, some SDS without displayed licenses were said by the PPB to be in the ‘registration process’, and could therefore neither be classified as registered or unregistered.

A number of policy questions emerge from the study. First, are all the regulations relevant for all SDSs in terms of their impact on public health and safety? For instance, is a refrigerator necessary for an SDS stocking medicines that do not require refrigeration? Is a narcotics cupboard necessary for an SDS not stocking narcotics, given that this is very rare in rural areas [46]? Is it really detrimental to health outcomes to stock a wider range of commodities beyond those that are medically-related? While these regulations are considered ideal for SDSs offering comprehensive pharmacy services, some may not be relevant for those offering a limited range of commodities, especially those in remote areas with low business potential.

In addition, SDSs operating in such areas are unlikely to attract staff with pharmacy qualifications, whose numbers are generally low in Kenya and other Sub-Saharan African countries as described above. This therefore raises the question of whether certain non-pharmacy cadres such as nurses or even nurse aides can be legally allowed to operate SDS in resource poor settings in order to maintain some access to medicines where there are few other reliable sources.

These questions suggest the need for considering “levels of practice” in the retail pharmaceutical sector in Kenya, that recognize resource-constraints and low business activity for operators working in remote areas. Under such a system, a lower level of SDS can be licensed by the medicine regulator to provide a limited range of medicines while being operated by individuals with some health-related training, and meeting a set of regulations appropriate to their product mix. This will require action by Kenyan stakeholders to discuss and identify practices and regulations for each level, involving reflection on the public health importance of each regulation. Such a system would require practice areas to be well-defined to avoid compromising the governance of pharmaceutical staff-operated SDSs in more urban locations, and to ensure that such lower level practices are only permitted in areas with access problems to alternative facilities. Already, countries like Tanzania, Ghana and Nigeria are implementing system of levels of practice for instance through the accredited drugs dispensing outlets (ADDOs) in Tanzania, licensed chemical sellers in Ghana, and patent medicine vendors in Nigeria [47–49]. The potential benefits of such a system are that it encourages rural operators to seek legitimization, and subscribe to agreed minimum standards, rather than either remaining unlicensed and unregulated. It also avoids a situation where there is routinely a high level of discordance between regulatory standards and implementation, which provides high potential for corruption. Qualitative work with SDS and regulatory inspectors demonstrated that the current discordance led to routine payment of bribes and great uncertainty for SDS operators [28].

Future research should explore ways of collecting information on regulatory factors that involve interaction with clients, particularly those that link directly to patient safety. This would require identifying aspects of regulation that can be measured using direct observation of operator-client interactions or use of mystery shoppers, which have been widely used in assessing ‘quality of care’ and some aspects of regulatory compliance [24, 50–52]. Studies should also examine knowledge of regulations in greater depth to gauge the degree to which non-compliance with regulation reflects poor knowledge or discordances between knowledge and practice, thereby showing the degree to which educational interventions would potentially be beneficial. Also required, is an in-depth understanding of the relationship between regulatory enforcement and the regulatory practices observed. This paper showed that regulatory compliance was poor, despite SDSs receiving regulatory visits. Future work should explore why compliance remains poor despite frequent regulatory inspections.

## Conclusion

The study set out to describe regulatory compliance among specialized drug shops in Kenya, and assess patterns of association between regulatory practices and selected predictors. Regulatory compliance was poor overall, but especially across rural shops, where the majority failed to meet requirements for staff qualification, structure, premises or equipment. Although regulatory inspections were common across both rural and urban locations, compliance was not influenced significantly by the frequency of such visits.

The poor regulatory compliance, particularly among rural operators, suggests a need for alternative approaches to regulating SDSs. Policymakers need to reexamine the list of regulations and decide whether all requirements should apply across the board, or whether regulatory requirements should be linked to the scope of practice and the location of the practice. Such an approach may reduce the number of SDSs operating unlawfully, and allow more shops to seek formal registration. Future research should focus on understanding reasons for poor regulatory compliance, especially among rural operators, despite what appear to be high frequencies of regulatory inspection.
